# What Constitutes Protective Immunity Following Yellow Fever Vaccination?

**DOI:** 10.3390/vaccines9060671

**Published:** 2021-06-18

**Authors:** Jolynne Mokaya, Derick Kimathi, Teresa Lambe, George M. Warimwe

**Affiliations:** 1 Centre for Tropical Medicine and Global Health, University of Oxford, Oxford OX1 3SU, UK; DKimathi@kemri-wellcome.org (D.K.); george.warimwe@ndm.ox.ac.uk (G.M.W.); 2 KEMRI-Wellcome Trust Research Programme, P.O. Box 230-80108, Kilifi 8010, Kenya; 3 The Jenner Institute, University of Oxford, Oxford OX3 7DQ, UK; teresa.lambe@ndm.ox.ac.uk

**Keywords:** yellow fever virus, yellow fever, yellow fever vaccine, humoral immune response, cell-mediated immune response

## Abstract

Yellow fever (YF) remains a threat to global health, with an increasing number of major outbreaks in the tropical areas of the world over the recent past. In light of this, the Eliminate Yellow Fever Epidemics Strategy was established with the aim of protecting one billion people at risk of YF through vaccination by the year 2026. The current YF vaccine gives excellent protection, but its use is limited by shortages in supply due to the difficulties in producing the vaccine. There are good grounds for believing that alternative fractional dosing regimens can produce strong protection and overcome the problem of supply shortages as less vaccine is required per person. However, immune responses to these vaccination approaches are yet to be fully understood. In addition, published data on immune responses following YF vaccination have mostly quantified neutralising antibody titers. However, vaccine-induced antibodies can confer immunity through other antibody effector functions beyond neutralisation, and an effective vaccine is also likely to induce strong and persistent memory T cell responses. This review highlights the gaps in knowledge in the characterisation of YF vaccine-induced protective immunity in the absence or presence of neutralising antibodies. The assessment of biophysical antibody characteristics and cell-mediated immunity following YF vaccination could help provide a comprehensive landscape of YF vaccine-induced immunity and a better understanding of correlates of protective immunity.

## 1. Introduction

Yellow fever (YF) is a disease caused by YF virus (YFV) which is known to cause death in about 30–60% of those infected [1,2]. The global annual prevalence of YF infection among humans is estimated at 200,000, with most cases reported in sub-Saharan Africa and South America where it is endemic [3]. Over the past five years, there have been outbreaks of YF in Brazil, Angola, Democratic Republic of Congo and Nigeria, with the risk of further spread to other countries and continents [4]. This global threat led to the establishment of the Eliminate Yellow Fever Epidemics (EYE) Strategy, steered by the World Health Organisation (WHO), United Nations Children’s Fund (UNICEF) and Gavi, the Vaccine Alliance [5]. The EYE strategy aims to protect one billion people against YF through vaccination by the year 2026 [5].

## 2. Molecular Biology of YF

**Figure 1:**
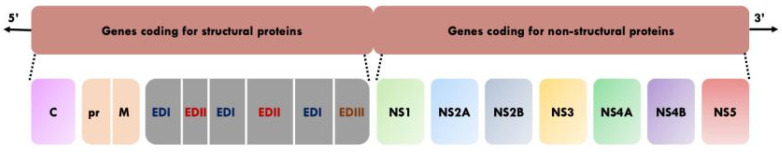
Genome organisation of YFV [2,7,8]. C: Capsid. pr: Precursor. M: Membrane. ED1: envelope domain I. EDII: Envelope domain II. EDIII: Envelope domain III. NS: Non-structural protein.

YFV is a member of the family Flaviviridae and genus *Flavivirus.* This genus includes other human and veterinary pathogens such as Dengue virus (DENV), Zika virus (ZIKV), tick-borne encephalitis virus (TBEV), West Nile virus (WNV) and Japanese encephalitis virus (JEV) [6]. The mature infectious virion is composed of an outer envelope made of a lipid bilayer derived from host membranes, and studded with dimers of envelop (E) glycoprotein and membrane (M) protein [2,7]. The E protein consists of three distinct domains (I, II, and III). Domain I links domains II and III, domain II facilitates viral attachment to target cells and fusion of the viral and host cell membrane, and domain III is thought to be involved in cell receptor binding [8]. The envelope surrounds the capsid (C) enclosing the viral genome which is a single-stranded positive sense RNA of approximately 11 kilobases [2,7]. The viral genome has a single open reading frame that encodes three structural proteins (E, C and M) which constitute the viral particle, and seven non-structural proteins (NS1, NS2A, NS2B, NS3, NS4A, NS4B and NS5) that are involved in viral RNA replication, virus assembly and modulation of host cell responses [2,7] (Figure 1).

Upon infection, the viral E protein facilitates entry of the virus into the target cell via receptor-mediated endocytosis, although the specific receptors remain unknown [8,9]. The endosomes containing the virus are then trafficked within the cytoplasm and the acidic environment of the endosomes triggers major conformational changes in the E protein resulting in the fusion of the viral and endosome membranes, thus releasing the viral genome into the cytosol [8,9,10]. Translation and processing of viral proteins are accomplished by host signalases and virus encoded protease (NS2B/NS3), respectively [9]. The RNA-dependent RNA polymerase (NS5) copies complementary negative-strand RNA from genomic RNA, which serves as a template for the synthesis of new positive-strand viral RNA [11]. Virion assembly begins in the rough endoplasmic reticulum whereby immature non-infective particles are formed. These particles contain M protein precursor (prM) and E glycoprotein forming spike-like trimers on the viral surface [12]. Viral maturation then occurs within the Golgi apparatus where the prM is cleaved, yielding an infectious viral particle with M and E proteins on the surface [12]. The release of progeny virions occurs within 10–12 h post-infection [11].

While both structural and non-structural YF viral proteins are antibody targets, neutralising antibodies (nAbs) are exclusively directed towards prM/M and E proteins, with E protein considered to elicit a stronger nAbs response compared to prM/M [8]. In addition, it has been shown that a large fraction of nAbs responses specifically target domain I and/or domain II of the E protein [13]. Protein similarities across human flaviviruses can result in the production of cross-reactive antibodies. For example, prior vaccination with inactivated Japanese encephalitis enhances YF immunogenicity after YF vaccination [14]. However, YF vaccine produces few flavivirus cross-reactive antibodies with only about 6% of YFV E-reactive monoclonal antibodies showing cross-reactivity to one or more heterologous flavivirus E protein [15].

## 3. Diversity and Transmission of YFV

**Figure 2:**
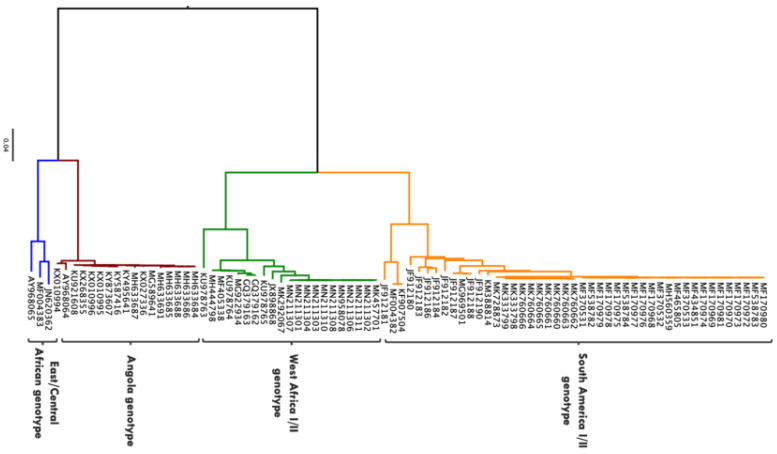
Phylogenetic tree showing 86 complete genome sequences of YFV isolated from biological samples obtained from human beings. Sequences obtained from Virus Pathogen Resource (https://www.viprbrc.org accessed on 26 May 2021). Maximum likelihood phylogenetic tree generated in IQ-TREE using the general time reversible nucleotide substitution model with gamma-distributed among-site rate variation (GTR + G) [20]. Phylogenetic tree rooted and visualised using FigTree program (http://tree.bio.ed.ac.uk/software/figtree/ accessed on 26 May 2021).

Molecular phylogenetic studies have described YFV diversity based on nucleotide sequence analysis of the whole genome as well as different sub-genomic regions [16]. It has been reported that the evolutionary rate of YFV, which is estimated at 2–5 × 10^−4^ substitutions/site/year, is generally consistent across different sub-genomic regions and therefore a representation of that of the entire genome [16,17]. While the mode of replication, fidelity of polymerase enzyme, ecology, epidemiology and immune response could play a role on the evolution of YFV, the specific drivers/mechanisms are yet to be determined [18]. There are seven major genotypes of YFV that have been described: five African (Angola, East Africa, East/Central Africa, West Africa I and West Africa II) and two South American (South America I and South America II) [16] (Figure 2). Based on complete genome sequences or sub-genomic sequences (M-E junction), the nucleotide difference between African and South American genotypes is up to 16%, whereas nucleotide difference between African genotypes is approximately 8% and approximately 5% between South American genotypes [16,19]. There are no studies that have investigated the relationship between YFV strains to specific phenotypes such as disease severity, possibly due to the availability of a vaccine that is effective across all strains resulting in limited interest in experimental investigations [16]. Nevertheless, the difference in geographical distribution of these genotypes can be attributed to a difference in the modes of maintenance and transmission. However, insufficient data are available to ascertain this [16].

**Figure 3:**
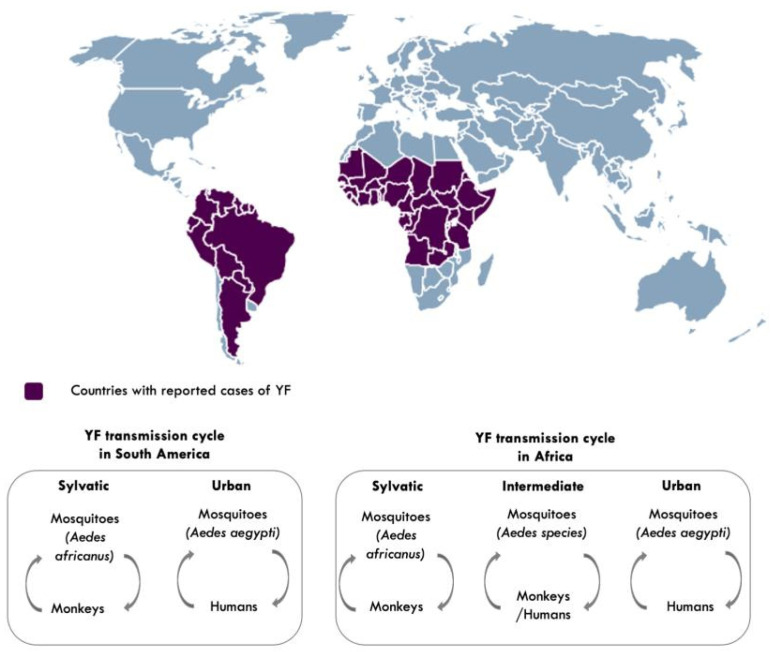
Countries with reported cases of yellow fever and YF transmission cycles [2,21]. Aedes species responsible for intermediate transmission include: *Aedes furcifer, Aedes luteocephalus, Aedes taylori, Aedes metallicus, Aedes vittatus, Aedes simpsoni complex.* Map made using (https://simplemaps.com accessed on 25 March 2021).

YFV is transmitted to humans and non-human primates in tropical areas of Africa and the Americas, via the bite of an infected mosquito [21]. Transmission to humans occurs in three cycles: sylvatic, intermediate, and urban [2,21] (Figure 3). In sylvatic transmission, infection to humans occurs when they enter forests where the virus is enzootically transmitted between non-human primates and mosquitoes. This sylvatic reservoir makes it challenging to eliminate YF [2,21]. In intermediate (savannah) transmission, humans residing in rural areas become infected when bitten by infected semi-domestic mosquitoes that feed on both humans and non-human primates, and in urban transmission, urban infected mosquitoes (*Aedes aegypti*) transmit the virus from human to human in densely populated areas [2].

## 4. Clinical Presentation, Diagnosis and Treatment of YF

**Figure 4:**
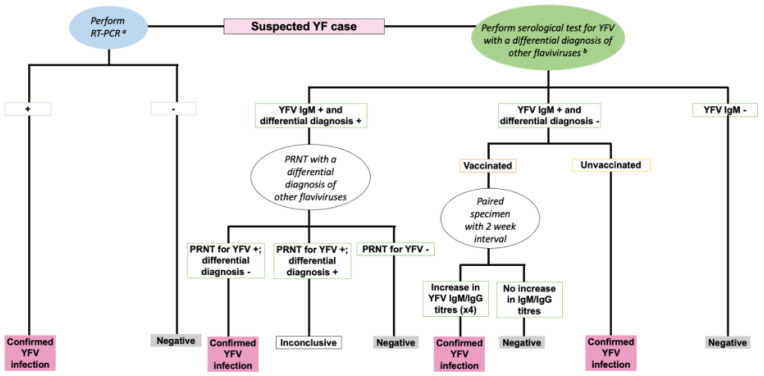
An algorithm for the diagnosis of YFV infection [22]. RT-PCR: Reverse Transcriptase Polymerase Chain Reaction. IgM: Immunoglobulin M. IgG: Immunoglobulin G. +: Positive. -: Negative. ^a^ RT-PCR performed on samples collected within less than 10 days of symptom onset. ^b^ Differential diagnosis of other flaviviruses such as DENV, WNV, ZIKV.

Most acute febrile infections are often self-limiting with viral replication occurring in regional lymph nodes [16]. However, approximately 20–50% of infected individuals develop pan-systemic sepsis characterised by viremia, fever, injury to the liver, kidney and heart, and haemorrhage [2,16]. Diagnosis of YF remains challenging given the differences in disease severity and symptom presentation in different infected individuals, similarity of clinical symptoms with other endemic diseases, and laboratory diagnosis which requires specialised resources that may not be accessible in areas where YF is endemic [2]. Furthermore, protein similarity of YFV to other flaviviruses (DENV, WNV, and ZIKV) often results in the production of cross-reactive antibodies thus making serological tests inconclusive [14,15,22]. Nevertheless, diagnosis can be made based using reverse transcriptase polymerase chain reaction to assess for YFV genomic RNA in body fluids, and/or using serologic tests which involve evaluating for the presence of YFV specific Immunoglobulin M (IgM) or Immunoglobulin G (IgG), with a differential diagnosis of DENV, WNV and ZIKV to rule out these viruses [2,22] (Figure 4). In addition, plaque reduction neutralisation antibody tests (PRNT) add specificity to the serological distinction by using a higher titre threshold (typically fourfold difference in PRNT titres) when comparing responses between flaviviruses [22]. There is no specific antiviral treatment for YF. However, early supportive clinical management of specific symptoms or complications (such as treatment for dehydration, fever, organ failure, and antibiotics for associated bacterial infections) could improve the outcome [2,16].

## 5. YF Prevention

There is a safe and effective vaccine against YF which was first developed in 1937 using a live attenuated YF virus strain (17D), with the subsequent production of YF vaccine using sub-strains (17D-204, 17DD and 17D-213) of 17D [1,23]. 17D was developed by passaging the virulent strain (Asibi) in rhesus macaques, mouse and chicken embryos causing mutations in genes encoding for both structural and non-structural proteins leading to the loss of its virulence [24]. The E protein of the 17D contains most of the mutations compared to other viral proteins, and given that E protein is responsible for viral attachment, fusion and is considered a major target for antibodies, mutations in this protein play a significant role in the attenuation of 17D [8,24].

The vaccine is administered intramuscularly or subcutaneously to adults travelling to endemic areas or periodically in response to outbreaks, and to children (>nine months of age) through routine childhood immunisation, with 80% and 100% of the vaccinees developing nAbs 10 days and one month post-immunisation, respectively [21,25]. There has been no difference reported in safety and protective immunity when the vaccine is administered either intradermally or subcutaneously [26]. Given evidence that the single primary dose of YF vaccine can provide lifelong immunity, a booster dose, which was previously given at an interval of 10 years from the primary dose, is no longer needed except among at-risk populations such as those who are immunocompromised or immunosuppressed [1].

Population YF vaccination coverage of >80% is recommended by the WHO to prevent and control outbreaks, however, YF vaccine coverage remains too low to prevent outbreaks especially in highly urbanised areas [5]. With the recent outbreaks, there is an increasing need to expand YF vaccine stocks since the current supply of YF vaccine is insufficient to provide effective coverage during outbreaks [1,4,21]. As a response, the WHO has recommended the use of fractional doses which have been used to control epidemics in Democratic Republic of Congo and South America, and studies have reported equivalent immunogenicity to that of the standard full dose [1,27,28]. However, immune responses to fractional doses of YF vaccine are yet to be fully understood. 

## 6. Quantity and Quality of YF Vaccine-Induced Immune Response 

YF vaccine induces several effector arms of the innate and adaptive immune response [29,30,31,32,33,34]. The early innate immune response to YF vaccine can offer protection from virulent virus and it also determines the strength and quality of adaptive immune response [35]. Upon vaccination, 17D infects dendritic cells (DC), where minimal transient viral replication occurs [35,36]. Multiple Toll-like receptors (TLR2, TLR7, TLR8 and TLR9) on these cells and their subsets (myeloid and plasmacytoid) become activated leading to the production of pro-inflammatory cytokines (including interferon-alpha) which induce an antiviral response, stimulates a mixed T helper 1- and T helper 2 cell profile, and regulate B cell responses [35,36]. DC also act as antigen presenting cells. They process and present internalised 17D epitopes to T cell receptors [35].

### 6.1. Cellular Immunity

**Figure 5:**
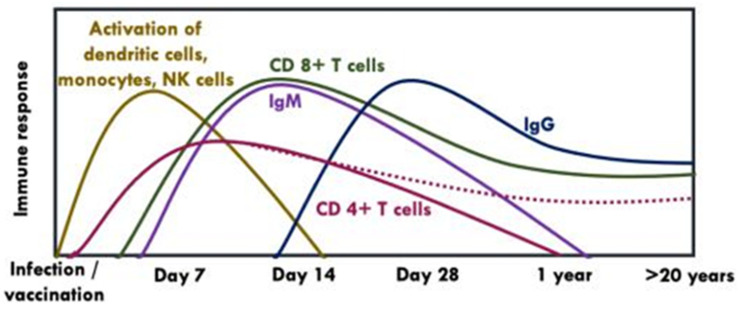
Kinetics of humoral and cellular immune response following vaccination with YFV-17D [38,39,41,42,43]. NK: Natural killer cells. The dotted line indicates that CD 4+ T cells may be present or absent in some individuals after years post-vaccination.

An effective vaccine is likely to induce antibodies as well as strong and persistent memory T cell responses [37]. Following antigen stimulation, CD4+ T cells produce cytokines which maintain CD8+ T cells and B cells whereas CD8+ T cells clear infected cells through cytolytic (via secretion of interferon (IFN)-γ and other cytokines) and non-cytolytic mechanisms (via perforin or Fas ligand-dependent apoptosis pathways) [37,38]. Unlike CD4+ T cells which have a slightly earlier appearance peaking between days 7 and 14 post-exposure but have a lower response to YFV-17D or natural infection, CD 8+ T cells peak at day 14 post-exposure and are stably maintained as memory CD 8+ T cells over decades [37], (Figure 5). Both CD4+ and CD 8+ T cells can recognise a broad array of epitopes within both structural and non-structural proteins of YFV [38,39,40].

A recent study reported that YFV specific memory CD 4+ T cells were present among unvaccinated individuals, however, with exposure to the virus/viral antigens, rare and more responsive T cells become recruited against the novel pathogen whereas pre-existing YFV-specific T cell populations with low clonal diversity undergo limited expansion [14]. Other studies have shown that YF vaccine elicits robust early effector CD 4+ T cell responses with YFV-specific memory T cells being readily detected in subjects examined years after vaccination although with a wide range of frequencies (i.e., 0–100 cells per million CD 4+ T cells) [38,39,41,42,43]. 

After approximately 14 days post-vaccination, total CD 8+ T cells become activated, undergo clonal expansion and differentiate to effector CD 8+ T cells which are distributed throughout the body to control for infection [38,44]. Effector CD 8+ T cells then differentiate to central memory and effector memory T cells (i.e., within four weeks post-vaccination) and remain detectable for decades [38,44], corroborated by a study which reported the possibility of a sufficient long-term immunity after vaccination given the presence of functionally competent YF-specific memory T-cell pool 18 years post-vaccination [29]. Similarly, memory T cells remained detectable eight years post-vaccination with fractional doses of YF vaccine and these markers of cellular immunity positively correlated with nAbs levels. These cellular immune responses elicited by the fractional dose were at comparable levels to those elicited by the standard full dose of YF vaccine [45]. There have been other conflicting data on cellular immunity to YF vaccine. For example, studies have reported a decline in the level of effector memory CD4+, CD8+ T cell, and interferon- γ+ CD8+ T cells after primary vaccination, suggesting the need for booster vaccination [30,46]. 

### 6.2. Humoral Immunity 

IgM mediates the early memory B cell response, which appears ~7–14 days following primary vaccination and can be detected up to 1–4 years post-vaccination [47]. Persistence of IgM has been linked to earlier onset viraemia or higher nAbs titers [35,47]. On the other hand, IgG develops slowly (i.e., within the first month of vaccination) and can last up to 40–60 years post-vaccination [15,35], (Figure 5).

A recently published review has summarised humoral immunity in adults and children who have received full dose vaccination [1]. Seropositivity rates among adults was >90% and ranged between 67% and 97%, within five years and ≥10 years post-vaccination, respectively, whereas in children, seropositivity rates ranged between 87% and 100% and between 28% and 76% within the first year and within ≥1–10 years post-vaccination, respectively [29,32,33,34,48,49,50,51,52,53]. Compared to adults, children seroconvert at a lower rate and have a larger decline in nAbs titres over the years suggesting the need for booster dose of YF vaccine in this age group. Nevertheless, the data available is scarce, and these findings cannot be generalised as the studies analysed were limited and heterogenous with respect to how samples and vaccines were handled, the type of vaccine strain used, and different seropositivity cut-off points employed [1,31]. With regard to immunogenicity following vaccination with fractional doses of YF vaccine, participants who received 1/100th, 1/50th, 1/10th, 1/5th and 1/3rd of YF vaccine doses had seroconversion rates of ≥87%, ≥92%, ≥97%, ≥95% and ≥98%, respectively, which lasted between eight and 10 years post-vaccination. These seroconversion rates were relatively similar to those of participants receiving the standard full dose which was at ≥95% [54].

nAbs are considered the primary correlate of protection following YF vaccination. Published data on immune responses following YF vaccination have only quantified YF virus-specific nAbs using either microneutralisation test for detection of antibodies or PRNT, reporting either 90% PRNT, 80% PRNT, or 50% PRNT titers, with a titre of 1 in 10 or higher considered a surrogate of protection [1,31]. While PRNT and microneutralisation assays are essential in evaluating antibody titre and neutralisation activity post-vaccination, they only assess limited humoral characteristics [55]. Furthermore, it has been shown that some vaccinated individuals who do not develop nAbs can develop secondary immune response with re-vaccination or exposure to infection [56,57]. 

The simultaneous binding of the fragment antigen-binding (Fab) regions of antibodies to foreign antigens expressed on the surfaces of pathogens or infected cells, and of the fragment crystallizable (Fc) portion of the antibody to Fc gamma receptors (FcγRs) that are expressed by immune cells, trigger antibody effector functions that eliminate pathogens such as antibody dependent cellular cytotoxicity (ADCC), antibody dependent cellular phagocytosis (ADCP), and antibody dependent complement deposition (ADCD) [58] (Table 1). Antibodies with these functions may or may not have neutralising activity and can recognise other pathogen proteins that are not involved in host-cell entry [55]. A study assessing the immune response in mice vaccinated with a chimeric Japanese Encephalitis vaccine (JE-CVax) and challenged with lethal YFV, showed that JE-Cvax could induce YFV-specific antibodies mediating ADCC in a dose-dependent manner [59]. Nevertheless, there are no studies that have characterised YF vaccine-induced antibody effector function in humans.

The capacity of antibodies to induce effector functions is also dependent on antibody isotype, subclass and glycosylation [55]. Some of the vaccine-induced polyclonal antibodies could either work collaboratively resulting in a more functional Fc effector profile or may compete against each other thus hindering Fc effector functions. This has been described in HIV vaccine trials where VAX003 vaccination resulted in elevated levels of IgG4 subclass antibodies, which have weak immune responses, competing for antigen occupancy thus blocking Fc effector functions, whereas RV144 vaccination resulted in elevated levels of IgG3 subclass antibodies, which elicited strong immune responses and also had the capacity to induce ADCC, ADCP and antibody-mediated activation of NK cells [55]. Antibody glycosylation determines specific antibody effector functions by altering the structure of antibody Fc region (through the addition of N-gylcan at specific asparagine residues on the Fc section) [60,61]. Majority of infection-associated immune profiling have focused on IgG Fc glycosylation and there is evidence to show that IgG Fc glycosylation can be altered following influenza and tetanus vaccination, and thus plays a critical role in shaping protective immunity [60].

While YF vaccine is considered highly successful with high seroconversion rates, there are other factors that have been associated with lower seroconversion rates or vaccine failures. These include age (i.e., premature waning of protection among vaccinated infants between the ages of nine and 12 months and the elderly >60 years), exposure to other childhood vaccines such as measles, mumps and rubella (MMR) and geographical regions especially YF endemic countries [1,66,67]. A study showed that after vaccination with YF-17D, individuals living in endemic areas had impaired immune responses with decreased persistence compared to those living in non-endemic areas [67]. 

## 7. Research Gaps

There are no studies that have provided a detailed characterisation of YF vaccine induced protective immunity in the absence or presence of nAbs. As demonstrated in studies evaluating Malaria, HIV and SARS-CoV-2 vaccine candidates or immune responses following infection, apart from neutralisation, antibodies can also engage FcγRs or the complement system to induce a range of Fc-effector functions which have robustly predicted protection from infection [55,68,69].

Systems serology for evaluation of vaccine-induced immune responses is not done routinely despite its ability to provide a comprehensive approach to assess the diversity of humoral immune responses, which can help inform vaccine development, delivery and dosing. The assessment of biophysical antibody characteristics following YF vaccination and associated risk factors including age, host genetics, geographical settings (i.e., endemic vs. non-endemic areas) and coinfections, could help provide a comprehensive landscape of the humoral immune response and a better understanding of correlates of protective immunity. 

Furthermore, with the current shortage of YF vaccine and the drive towards using fractional dosing—whose evidence has been based solely on quantified titers of YF virus-specific neutralising antibodies [54]—data evaluating YF vaccine-induced cellular immunity using this dose regimen are needed. T cell and memory B cell responses have been shown to be predictive of the quality and quantity of the humoral immune response. However, it remains unclear whether and how cellular immunogenicity is protective against YF virus infection. Data describing the magnitude and duration of cellular immune response especially with fractional vaccine doses as well as the correlation between cellular and humoral immune response following this vaccination regimen are needed. In addition, a better understanding of both humoral and cellular immunity following vaccination might also help predict long-term immune responses without having to obtain data over long duration of follow-up. 

## Figures and Tables

**Table 1:** Summary of antibody effector functions [62,63,64,65].

Antibody Effector Function	Antibody Dependent Cellular Phagocytosis (ADCP)	Antibody Dependent Cellular Cytotoxicity (ADCC)	Antibody Dependent Complement Deposition (ADCD)
Description	Following activation of Fc receptors, effector cells eliminate antibody-opsonised pathogens through phagocytosis and also activate adaptive immune responses by facilitating antigen presentation and/or secretion of inflammatory mediators	Following activation of Fc receptors, effector cells recognise and kill antibody-coated target cells through perforin/granzyme cell death pathway, FAS-L pathway and/or reactive oxygen species pathway	Antibody-antigen complexes activate complement proteins which, following a cascade of enzymatic reaction, result in the assembly of membrane attack complexes and the formation of pores on the surface of target cells or pathogens causing cell-lysis
Antibodies/Fc receptors commonly implicated in specific antibody effector functions	IgA (FcαRI); IgG-dependent (FcγR1, FcγRII, FcγRIIIa)	IgG-dependent (FcγR1, FcγRII, FcγRIIIa)	IgM, IgG1, IgG2, IgG3, IgG4

Effector cells include: neutrophils, monocytes, macrophages, eosinophils, basophils and natural killer cells.

## Data Availability

No new data were created or analysed in this study. Data sharing is not applicable to this article.
